# Computerized adaptation and validity evidence of the test of assessment of syntactic processing in comprehension (TASComp) in Brazilian adults

**DOI:** 10.3389/fnhum.2026.1791627

**Published:** 2026-03-20

**Authors:** Carolina Aguiar de Oliveira, Larissa Rangel Ferrari, Hellora Caroline Izidoro Rodrigues, Amanda Maciel Schlaepfer, Helenice Charchat Fichman, Erica dos Santos Rodrigues, Jaqueline Carvalho Rodrigues

**Affiliations:** 1 LabINS - Interdisciplinary Laboratory for Neurodevelopment and Health, Department of Psychology, Pontifical Catholic University of Rio de Janeiro (PUC-Rio), Rio de Janeiro, Brazil; 2 LAPAL - Psycholinguistics and Language Acquisition Laboratory, Department of Letters, Pontifical Catholic University of Rio de Janeiro (PUC-Rio), Rio de Janeiro, Brazil; 3 NEUROPSICLIN - Laboratory of Clinical Psychology Research, Department of Psychology, Pontifical Catholic University of Rio de Janeiro (PUC-Rio), Rio de Janeiro, Brazil

**Keywords:** evidence of validity, language assessments, psycholinguistics, reading comprehension, sentence comprehension, syntax

## Abstract

**Introduction:**

Instruments for the assessment of syntax in Brazilian Portuguese remain scarce, despite their crucial role in identifying comprehension and production deficits in clinical populations. This study aimed to develop a computerized version of the Test for the Assessment of Syntactic Processing in Comprehension (TASComp) and to investigate validity evidence related to sentence reading comprehension among Brazilian adults.

**Methods:**

Procedures included stimulus adaptation for the PsyToolkit platform, content validation by expert judges, and psychometric analyses of a final version administered to 102 healthy adults, stratified by in-person (*n* = 45) and online (*n* = 57) administration modalities.

**Results:**

The TASComp demonstrated satisfactory content validity, with a Scale Content Validity Index (S-CVI) of 0.87 and adequate internal consistency (KR-20 = 0.66). Syntactic complexity effects were confirmed by significant differences in the results of reversible passives vs. irreversible (*t* = 3.08, *p* = 0.003, *d* = 0.31), object clefts vs. subject clefts (*t* = 5.30, *p* < 0.001, d = 0.53), and center-embedded vs. right-branching relatives for both subject clauses (*t* = 6.80, *p* < 0.001, *d* = 0.67) and object clauses (*t* = 3.52, *p* = 0.001, *d* = 0.35). Additionally, right-branching subject relatives were more accurate than right-branching object relatives (*t* = 3,78; *p* < 0.001, *d* = 0.37). No significant differences in accuracy were found between in-person and online administration modalities across all sentence types (*p* > 0.05). The beta regression model revealed that years of education (χ2 (1) 35 = 15.74, *p* < 0.001) and age (χ2 (1) = 7.98, p = 0.01) were significant predictors of comprehension accuracy, while the administration modality was not (χ2 (1) = 0.27, *p* = 0.61).

**Discussion:**

Validity evidence based on response processes was observed, as the instrument successfully discriminated between levels of syntactic complexity, with significant performance differences in structures that impose high working memory demands, such as object clefts and center-embedded and object relative clauses. No significant differences were found between administration modalities (in-person vs. online), indicating that both modes assess sentence reading comprehension without compromising psychometric quality. Furthermore, the findings demonstrate that age and years of education significantly impact syntactic processing and reading comprehension.

**Conclusion:**

TASComp demonstrates adequate validity of evidence and clinical relevance. The instrument shows promise for detecting subtle language deficits that are often overlooked by other assessments, due to its inclusion of a wide range of 46 complex syntactic structures.

## 1 Introduction

Syntactic processing encompasses the set of cognitive operations that support the incremental construction of grammatical structures during language comprehension and production (Blake et al., 2025; Caplan et al., 2016; Rodrigues et al., 2024). This process entails the hierarchical organization of constituents, the computation of dependency relations, and the integration of syntactic and semantic information in real time (Breder and Rodrigues, 2026; Caplan, 2016). Disruptions to these mechanisms may result in sentence-level comprehension deficits (Rodrigues et al., 2024).

Language assessments, specifically at the syntactic level, are highly sensitive to neurocognitive disorders and early changes associated with cognitive decline and dementia in adults (Gilardone et al., 2024; Ivanova and Dronkers, 2022; Kaltsa et al., 2024; Sung et al., 2020). In adult neurodevelopmental disorders, such as Attention-Deficit/Hyperactivity Disorder (ADHD) (Hill et al., 2025; Jepsen et al., 2025; Oliveira and Corrêa, 2023; Parks et al., 2022) and Developmental Language Disorder (Bishop et al., 2017; Blake et al., 2025; Corrêa and Augusto, 2013; Tucci and Choi, 2023), syntactic production and comprehension may likewise be impaired, with significant deficits manifesting at both sentential and discursive levels. Consequently, the assessment of syntactic processing should not be restricted to aphasic populations; rather, it warrants inclusion in comprehensive investigations of syntactic and semantic interface across a broad spectrum of adult clinical cohorts (Gilardone et al., 2024; Ivanova and Dronkers, 2022; Kaltsa et al., 2024; Murray, 2018; Thothathiri et al., 2023; Wallace et al., 2023).

The diagnostic value of syntactic assessments lies in the high processing demands of complex structures, whose performance is affected not only by linguistic deficits but also by reduced working memory capacity (Caplan et al., 2016; Murray, 2018; Poulisse et al., 2019; Sung et al., 2017). Although linguistic processing remains stable in aging (McKoon and Ratcliff, 2018; Silagi et al., 2023; Solomons et al., 2025), syntactic difficulties in processing complex sentences may be present due to decreased executive functions (Murray, 2018; Poulisse et al., 2019; Sung et al., 2020). These deficits, even in healthy aging, can serve as preclinical indicators of cognitive difficulties (Feigin et al., 2017; Kaltsa et al., 2024; Van Oers et al., 2018). Furthermore, education and lifelong literacy can serve as a protective factor for linguistic performance against cognitive decline (López-Higes et al., 2023; Riffo et al., 2025). These factors, essential for maintaining linguistic abilities (Pereira and Ortiz, 2022), can also stimulate the attentional and executive resources necessary for processing complex sentences (Akashi and Ortiz, 2018; Malcorra et al., 2022).

Sentence comprehension assessments must be grounded in psycholinguistic parameters that identify syntactic configurations associated with increased processing cost. Such costs are primarily linked to deviations from the canonical Subject–Verb–Object (SVO) order and to structures involving syntactic displacement (Rodrigues et al., 2024). Passive sentences exemplify this deviation by promoting the object of the corresponding active clause to subject position (“The fence was painted by the worker”; *A cerca foi pintada pelo funcionário* [Brazilian Portuguese, pt-BR]) and by demoting or omitting the agent (“The worker was fired last week”; pt-BR: O funcionário foi demitido *na última semana), which disrupts the canonical mapping between thematic roles and grammatical*), functions and increases reliance on syntactic parsing (Corrêa et al., 2017; Sung et al., 2020). Processing difficulty is amplified in reversible passives, where animacy does not constrain thematic role assignment (“The man was greeted by his friend”; pt-BR: *O homem foi cumprimentado pela sua amiga*); and in cases of passives with implausible semantic content (“The police officer was chased by the criminal”; pt-BR: *O policial foi perseguido pelo bandido*), which limit the use of semantic heuristics and force greater dependence on structural cues (Christianson et al., 2006; Ferreira et al., 2002). These demands are particularly taxing for populations that rely on good-enough processing strategies, such as older adults, who may prioritize semantic plausibility over detailed syntactic analysis, with evidence showing higher processing costs for passives in Brazilian Portuguese within these groups (Ferrari, 2017; Hübner et al., 2022; Souza, 2023).

Additional processing costs arise in constructions that involve filler–gap dependencies, such as cleft sentences and relative clauses. Cleft sentences consist of a matrix clause, typically introduced by the copular construction “it is” (in Brazilian Portuguese, “*É*”), and a relative clause that encoded the dependency associated with the focused constituent (Alves et al., 2015). The complexity of these sentences lies in the syntactic movement of the focused argument, leaving a trace in its original position (“It is the girl [who __ is helping the woman]”; *É a menina que está sendo ajudada pela mulher* [BP]) (Alves et al., 2015; Lambrecht, 2001). Relative clauses involve the movement of a constituent to an antecedent position in the sentence, leaving a gap in its original location immediately following the relative pronoun (The boy [who__ honored the teacher] is an excellent student; O menino que elogiou o professor é um excelente estudante). Object clefts and object relative clauses are consistently more demanding than their subject counterparts due to the greater linear and structural distance between the displaced constituent and its gap, increasing working memory load and susceptibility to interference (Forster and Corrêa, 2016; Friedmann et al., 2009; Gibson, 1998). Center-embedded relative clauses further exacerbate this cost by interrupting the main clause and requiring the maintenance of multiple unresolved dependencies, in contrast to right-branching structures, which impose lower processing demands (Gibson et al., 2005). Finally, sentences with perspective verbs introduce noncanonical mappings between syntactic position and thematic role, as the subject denotes a patient rather than an agent (“The patient received the diagnosis from the doctor”; pt-BR: *O paciente recebeu o diagnóstico do médico.*), increasing computational demands despite active syntax (Breder and Rodrigues, 2026). Together, these structures highlight the importance of controlling syntactic complexity when designing assessment tools sensitive to sentence-level processing deficits (Rodrigues et al., 2024).

Language assessments with different syntactic structures could be essential to identify deficits that compromise reading comprehension and the interpretation of complex messages, thereby affecting communicative competence in daily life. Although individuals often employ compensatory strategies, such as re-reading, slowing down, and minimizing distractions, processing deficits tend to surface specifically under conditions of increased syntactic complexity (Webster et al., 2023). These difficulties can significantly interfere with modern reading habits, particularly in the realm of digital communication, such as instant messaging, which requires the rapid processing of sentence structures (Kinsey et al., 2021; Lee and Cherney, 2022). In clinical settings, detailed syntactic assessment serves as a sensitive screening tool for subtle linguistic impairments that frequently go undetected in spontaneous conversation or simple comprehension tasks (Sung et al., 2020). Consequently, the heightened cognitive effort required for language processing can lead to reduced social participation, affecting both elderly populations and adults with acquired language disorders who rely on technology for interpersonal connection (Kinsey et al., 2021). Ultimately, a comprehensive evaluation of syntax is also fundamental for designing interventions in clinical settings, considering a detailed evaluation of language comprehension.

Despite robust evidence of deficits associated with high-cost syntactic structures, such as passive, relative, and cleft sentences, in clinical and aging populations, standardized instruments for evaluating sentence comprehension remain scarce in Brazil. Current assessment options primarily include the adapted versions of the Montreal-Toulouse Battery (MTL-BR) (Parente et al., 2016) and the Test for Reception of Grammar (TROG-2) (Carthery-Goulart et al., 2022; Pereira et al., 2009). Both instruments provide essential validity evidence for the Brazilian population but present some limitations. The MTL-BR, which evaluates different levels of language processing, includes only eight items assessing written sentence comprehension. By contrast, the TROG-2, designed to evaluate oral syntactic comprehension, exhibits an imbalance in the representation of sentence subtypes (e.g., discrepancies between reversible and irreversible items in blocks) and variability in constituent features, such as animate versus inanimate subjects. This stimulus heterogeneity within blocks may confound qualitative error analysis and hinder the precise assessment of discrete syntactic structures (Ferrari, 2022).

In this context, the Test for the Assessment of Syntactic Processing in Comprehension (TASComp) was developed (Ferrari, 2022). Designed in Brazilian Portuguese, the instrument employs age-appropriate stimuli depicting human agents performing various actions. Importantly, TASComp includes semantically implausible sentences to require the inhibition of good-enough processing strategies (Frances, 2024), thereby limiting reliance on semantic heuristics and ensuring that performance depends on syntactic parsing (Ferrari, 2022).

The TASComp was constructed based on psycholinguistics theory about processing cost of sentences (Ferrari, 2022). Regarding its structure, TASComp comprises five blocks, each representing a specific syntactic construction: active, passive, cleft, relative, and interrogative clauses. These blocks include subcategories reflecting varying levels of linguistic complexity, totaling 56 stimuli and encompassing 14 sentence types: subject and object relative clauses (in both right-branching and center-embedded forms); subject and object cleft sentences; reversible passives (classified as neutral or implausible) and irreversible passives; constructions with perspective verbs; simple active sentences (reversible and irreversible); and subject and object interrogatives. TASComp includes four items per sentence type.

The original TASComp version was designed for stimulus presentation using PowerPoint. Each trial presents a target sentence alongside a quadrant containing four image-based response options (A, B, C, and D). By keeping the sentence visible throughout the visual array, this design reduces working memory demands during syntactic processing (Tan et al., 2017), allowing for a more precise assessment of domain-specific linguistic impairments. Responses were recorded on a printed protocol, completed either by the participant during group administration or by the examiner in individual sessions (Ferrari, 2022).

The assessment score considers one point per correct response, which permits both a quantitative evaluation of cumulative accuracy in each block and in the total score of the task (Ferrari, 2022). Furthermore, the TASComp incorporates an analysis of error patterns to elucidate the nature of underlying linguistic impairments based on the respondent’s selections. Grammatical errors are characterized by thematic role of reversals, such as agent-patient inversion, which indicate specific syntactic parsing failures. Lexical errors involve the substitution of core lexical constituents, such as the selection of an alternative verb, while maintaining an interpretation consistent with the original thematic structure. Finally, mixed errors manifest as the simultaneous alteration of both syntactic configuration and lexical content, reflecting a multidimensional impairment across various domains of linguistic processing (Ferrari, 2022).

The applicability of TASComp was evaluated in 43 adults aged 18 to 57 years (*M* = 23.41, *SD* = 8.76) and 10 older adults aged 60 to 74 years (*M* = 65.50, *SD* = 5.17), all with a university degree (Ferrari, 2022). In the adult group, the mean number of correct responses was 54.63 out of 56 sentences (approximately 97.50%), with eight participants making errors on relative clauses (the structure with the highest error rate). The older adult group achieved a mean of 51.60 correct responses (approximately 92.40%), with errors predominantly occurring in relative clauses and across the test as a whole (Ferrari, 2022). However, the small sample of older adults (*n* = 10) limited the number of comparisons that could be made between groups.

Although TASComp is a promising tool for assessing adults’ syntactic comprehension, its psychometric properties have not yet been analyzed. Developing a computerized version would enable administration in both face-to-face and remote/online settings, supporting teleneuropsychology and interdisciplinary applications (Alva et al., 2025; Serrano-Juárez et al., 2023). In this context, it is essential to validate the test for online use and to examine whether its psychometric properties differ from the traditional pencil-and-paper version. Current research indicates strong correlations between remote and in-person assessment results, with differences generally lacking significant clinical impact across most tests analyzed (Brown et al., 2024; Chapman et al., 2021; Krynicki et al., 2023).

This study aimed to adapt TASComp to a computerized online version and to investigate its psychometric properties. The specific objectives were: (a) to design the computerized version of TASComp, (b) to analyze evidence of content validity through expert judgment, (c) to test the instrument in a pilot study, and (d) to examine evidence of validity based on internal consistency, response processes across administration modalities (in-person and online) and syntactic complexity, as well as relationships with external variables (age and years of education) in each modality. Overall, the study seeks to contribute a Brazilian computerized instrument for assessing the syntactic level of written comprehension in adults.

## 2 Materials and methods

The computerized version of TASComp was developed in six stages: (1) adaptation of stimuli and data-recording procedures; (2) expert review; (3) revisions; (4) pilot study; (5) refinement of administration instructions; and (6) analysis of the psychometric properties of the final version. Each stage involved distinct participants and procedures, as detailed below. The study was approved by the institutional ethics committee (approval no. 7.719.167).

### 2.1 Step 1: stimulus adaptation for computerized version of TASComp

At this stage, the TASComp stimuli was designed into the PsyToolkit (Stoet, 2016). This platform was selected for its ability to present stimuli in an assessment design, record participants’ responses, and reaction times. The interrogative sentences block contained in the original version (Ferrari, 2022) was excluded due to the variability in stimulus presentation. The online version proposed contains 48 items organized into five sentence types: active (12 items), passive (12 items), relative (16 items), and cleft sentences (8 items). Two training stimuli were included. Table 1 shows examples of sentences from each of the TASComp blocks.

**Table 1:** Types of syntactic clauses in TASComp and examples of items in Brazilian Portuguese (pt-BR) and their translation into English.

Type of syntactic clause	Block	Example (pt-BR)	Example translated (EN)
Irreversible active	Active sentences	A menina está lendo o livro.	The girl is reading the book.
Reversible active	Active sentences	A menina está puxando o menino.	The girl is pulling the boy.
Sentences with perspective verb	Active sentences	O rapaz está ganhando o presente da moça.	The boy is receiving a gift from the girl.
Irreversible passives	Passive sentences	O quadro está sendo pintado pelo rapaz.	The painting is being painted by the boy.
Reversible passives	Passive sentences	A mulher está sendo consolada pela menina.	The woman is being comforted by the girl.
Semantic implausible reversible passives	Passive sentences	O repórter está sendo entrevistado pela médica.	The journalist is being interviewed by the doctor.
Object clefts	Clefts	É o soldado que o astronauta está empurrando.	It is the soldier that the astronaut is pushing.
Subject clefts	Clefts	É o menino que está abraçando o homem.	It is the boy who is hugging the man.
Right-branching subject relative	Relative clauses	O menino cutuca o homem que está quicando a bola.	The boy pokes the man who is bouncing the ball.
Center-embedded subject relative	Relative clauses	O rapaz que filma a moça está descendo a escada.	The boy that films the girl is going down the stairs.
Right-branching object relative	Relative clauses	A figura certa mostra o homem que a mulher está consolando.	The correct figure shows the man that the woman is consoling.
Center-embedded object relative	Relative clauses	A menina que a mulher observa está subindo o degrau.	The girl that the woman observes is going up the step.

Stimuli were randomized to minimize performance differences attributable to item difficulty, independent of block. An instruction manual was developed to standardize administration across evaluators, and task instructions were embedded in the computerized platform. Finally, a qualitative questionnaire was administered at the end of the task to assess participants’ response strategies and motivation.

### 2.2 Steps 2 and 3: content validity and reformulations

#### 2.2.1 Participants

In Step 2, 22 researchers from a completed item evaluation form administered via Google Forms, including undergraduate and graduate students in Psychology and Linguistics. The research participants consisted entirely of Brazilian graduate students from the state of Rio de Janeiro. To establish content validity, the Content Validity Index (CVI) aggregates expert ratings regarding item relevance and clarity (Yusoff, 2019). The index is derived from the proportion of experts endorsing an item’s essentiality. This quantitative validation is crucial for minimizing measurement error, ensuring that the instrument accurately operationalizes the intended theoretical construct (Lynn, 1986).

For the calculation of the CVI, data from seven judges meeting a minimum qualification of a master’s degree and affiliated with graduate programs in Clinical Psychology or Language Studies were considered. The panel comprised one master’s-level and two doctoral researchers in Literature and Language Studies with expertise in psycholinguistics, one doctoral researcher in Clinical Psychology, the panel was comprised of three master’s-level researchers in Clinical Psychology with experience in neuropsychological assessment and psychometrics.

#### 2.2.2 Materials and procedures

To assess the content validity of the online TASComp, judges reviewed all 48 items, each comprising the sentence and four image-based response options. For each item, they provided dichotomous (“Yes”/“No”) responses to two criteria: (1) whether the target image adequately represented the sentence content, and (2) whether the distractors were sufficiently differentiated from the target for accurate sentence interpretation. In the second section of the form, judges rated the test instructions, layout, and qualitative feedback questionnaire on a 4-point Likert scale (1 = totally inadequate, 4 = quite adequate), with space for additional comments.

#### 2.2.3 Statistical analyses

Microsoft Excel (Md Lukmanul Hakim et al., 2025; Microsoft Corporation, 2024) was used to calculate the indices according to Yusoff (2019) tutorial. For the calculation for the content validity index (CVI) Item and Scale-CVI (Yusoff, 2019), a score of 1 was assigned for “Yes” and zero for “No” for the dichotomous questions. The CVI was calculated as the proportion of agreement among the judges, with values above 0.83 considered acceptable recommended by Lynn (1986), considering six to eight experts’ evaluation. For Likert-scale responses, values of 1 and 2 were recorded as zero (inadequate), and 3 and 4 were recorded as 1 (adequate) for calculation purposes. The responses of undergraduate researchers were compiled for qualitative evaluation.

### 2.3 Steps 3 and 4: pilot study and final version design

#### 2.3.1 Participants

A pilot study was conducted with six brazilian adults, between 18 to 48 years, to evaluate the usability and functionality of the computerized TASComp after the item modifications. Participants were classified as cognitively healthy based on the self-reported absence of any history of psychiatric, neurological, or cognitive disorders in a clinical interview. The pilot study sample comprised Brazilian residents of Rio de Janeiro who were enrolled in undergraduate or Master’s degree programs. Following the pilot study, the computerized version of the TASComp was reformulated for the final version.

#### 2.3.2 Materials and procedures

A convenience sample was recruited via social media. After providing informed consent, participants completed a sociodemographic and health questionnaire, followed by the TASComp. Four participants were assessed online via Zoom, with the evaluator sharing their screen to present instructions, training, and test items. Two participants were assessed in person in the research laboratory, with the task displayed on the researcher’s computer. In both modalities, the test was administered by the researcher on a computer or laptop with a minimum 14-inch screen in a quiet environment, ensuring participant confidentiality; microphones and headphones were used in the online condition. Participants responded verbally by indicating the letter corresponding to their chosen option (A–D), which was recorded by the evaluator. Based on accuracy data and qualitative usability feedback, the final version of the TASComp was developed.

#### 2.3.3 Statistical analyses

In Step 3, analyses included computing each participant’s total number of correct responses and extracting results using RStudio and the Tidyverse package (R Core Team, 2025; Wickham et al., 2019), along with the collection of qualitative data. Qualitative information was obtained from evaluator–participant conversations and focused on feedback regarding the test’s design and interface, clarity of the final instructions, and overall platform usability from the evaluator’s perspective.

### 2.4 Step 5: psychometrics properties of the online version of TASComp

#### 2.4.1 Participants

Based on sample calculations for multiple linear regression analysis with two predictors, the minimum sample size required to conduct the study was 44 individuals, considering a probability of *α* = 0.05 and statistical power of 1−β = 0.95. The software used for the sample calculation was G*Power (Faul et al., 2007). The final testing sample comprised 102 Brazilian adults, all residents of Brazil from different states across the country, native speakers of Brazilian Portuguese with at least 5 years of formal education and no self-reported neurological or psychiatric diagnoses. Exclusion criteria included a history of self-reported neurodevelopmental disorders, psychiatric disorders without medical treatment or with partial symptom remission, illiteracy, and uncorrected hearing or visual impairments. Participants were divided into two groups: 45 completed the in-person assessment and 57 completed the remote assessment. Participants assessed online were younger than those assessed in person. A summary of the sample’s sociodemographic characteristics is presented in Table 2.

**Table 2:** Sociodemographic characteristics of the participants.

Variable	In-person (*n* = 45)	Online (*n* = 57)	Total (*n* = 102)	*t*/(χ^2^)	*p*
Age (years) *M* (SD)	51.20 (20.00)	42.6 (16.30)	45.8 (18.06)	2.65	0.009*
Years of study *M* (SD)	17.40 (4.75)	18.00 (4.05)	17.80 (4.36)	−0.64	0.53
Gender %					
Woman	24.50	33.30	57.80	0.17	0.68
Men	19.60	22.50	42.20		
Family income^a^ %					
A—21,826.74	12.70	16.50	29.10	5.00	0.42
B1—10,361.40	7.60	21.50	29.10		
B2—5,755.23	10.10	10.10	20.30		
C1—3,276.76	6.30	5.10	11.40		
C2—1,965.87	5.10	2.50	7.60		
D, E—900.60	1.30	1.30	2.50		

a Socioeconomic classification based on the possession of consumer goods and education level (Brazil Economic Classification Criteria/ABEP).

#### 2.4.2 Materials and procedures

The sample was non-probabilistic and convenience-based, recruited via university announcements, social media, and referrals. Data were collected individually by the principal researcher or by trained undergraduate students in Psychology and Neuroscience. Procedures followed those described in the pilot study. For online assessments, participants joined a Zoom video call, were expected to be familiar with technology or have assistance and viewed the evaluator’s shared screen. Both participants and evaluators were in a quiet, interruption-free environment, using headphones to minimize noise and ensure confidentiality.

In-person TASComp assessments were conducted on computers in research laboratories or private rooms. Participants sat beside the examiner, facing the screen at an appropriate distance for clear visibility. The examiner recorded the participant’s responses after they indicated their choice verbally or by pointing to the corresponding letter. Following the task, the examiner completed a qualitative questionnaire, documenting response mode and interaction, as described in the pilot study.

#### 2.4.3 Statistical analyses

Descriptive analyses of the sample were performed, including normality testing (Shapiro–Wilk) for sociodemographic variables (age and years of schooling) and TASComp performance, both overall and by block (Richardson and Machan, 2021). Group differences by administration modality (in-person vs. online) were assessed using Student’s t-test for age and years of schooling and the Chi-square test (χ^2^) for gender and family income. TASComp data were extracted using RStudio and the Tidyverse package (R Core Team, 2025; Wickham et al., 2019), recording correct responses and constructing dataframes for analysis in Jamovi (The Jamovi Project, 2025). Extracted data included item-level accuracy, total correct responses per participant, and correct responses by syntactic block. Means and standard deviations were calculated for each block and the total test score.

Evidence of validity based on internal structure was assessed using the Kuder–Richardson 20 coefficient (KR-20) (Kuder and Richardson, 1937; Zhang, 2022) for the full scale. Validity evidence based on response processes was examined by comparing performance differences associated with the structural complexity of each test block using paired-samples *t*-tests. Manipulated syntactic factors included: reversibility in active and passive sentences (reversible vs. irreversible); clause type in relative and cleft sentences (subject vs. object); relative clause position (center vs. right-branching); and semantic plausibility in reversible passive sentences. Additionally, mean performance per block across syntactic complexity levels was compared between administration modalities (in-person vs. online) using independent-samples *t*-tests. All *t*-tests were conducted in SPSS 27 (IBM Corp, 2020) with Bias-Corrected and Accelerated Bootstrap (BCa) resampling (1,000 samples) to ensure robust estimates (Field, 2017; Haukoos and Lewis, 2005).

To examine criterion validity, the influence of age and education on the total proportion of correct TASComp responses was analyzed, with administration modality (in-person vs. online) included as a factor. A Beta Regression Model (Schmid et al., 2013) was implemented in the GAMlj3 module of Jamovi (Gallucci, 2019) using a logit link function to model the overall proportion of accuracy (0–1) within a Generalized Linear Model framework. Beta regression is appropriate for dependent variables expressed as proportions, offering a more suitable alternative to linear regression for non-parametric data (Shapiro–Wilk < 0.001; Kerby, 2014). Robustness of parameter estimates was assessed via 95% confidence intervals using the BCa with 1,000 resamples (Haukoos and Lewis, 2005).

## 3 Results

### 3.1 Step 1: stimulus adaptation for online computerized version of TASComp

The initial phase of the adaptation process yielded the elaborated instructions specifically designed for the computerized version of TASComp. The computerized TASComp must be administered and recorded solely by the evaluator and is not suitable for self-administration; all test interactions are performed using the evaluator’s mouse. Both remote and in-person assessments require a quiet, well-lit, and interruption-free environment. Equipment requirements include a computer with a minimum 14.6-inch screen and an internet connection of at least 20 Mbps. The computer should be placed on a flat surface at eye level, maintaining an approximate viewing distance of 60 cm from the participant.

The computerized TASComp is administered in four sequential steps: (1) review of instructions with the participant, (2) two training items, (3) administration of the main test comprising 48 sentences, and (4) completion of a qualitative questionnaire by the evaluator. Designed as a silent reading task, participants indicate their responses from the training stage onward either verbally (stating the letter of the chosen answer) or pointing. Each item provides feedback on correctness, with a maximum of five errors allowed per training item; exceeding this limit results in test termination. Participants are informed that answers cannot be changed once submitted and are encouraged to respond promptly, as the test also measures processing speed (time per item).

**Figure 1:**
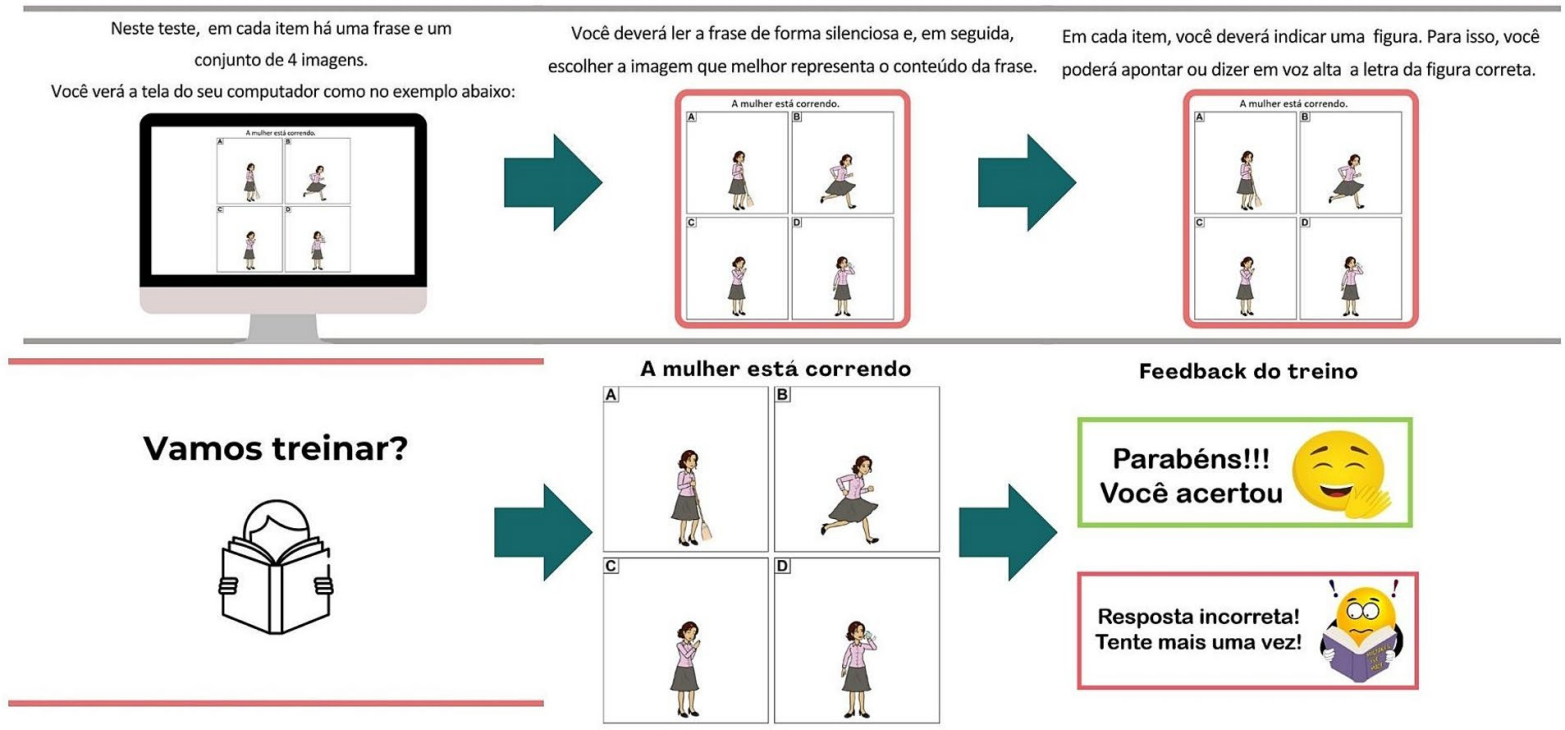
Computerized TASComp instructions and training example on the PsyToolkit platform.

The estimated duration of the TASComp is approximately 15 min, although there is no strict time limit for completion. An example of the steps preceding the training phase is shown in Figure 1.

Upon completion of the 48 items, a screen indicates the end of the task. The evaluator then stops screen sharing or removes the display from the participant’s view to complete a qualitative questionnaire for evaluator use only. The questionnaire records the participant’s primary response mode (verbal or pointing), including responses mediated by assistants if applicable. It also documents whether sentences were read aloud, instances of re-reading, and self-corrections (errors corrected before proceeding to the next item). Additionally, the evaluator notes the participant’s motivation, collaboration, and any relevant behavioral observations, providing essential clinical context for interpreting results.

The final TASComp version was published via a PsyToolkit link accessible only to study researchers. A sample link containing three test items was also created to train administrators on instructions and platform use.

### 3.2 Steps 2 and 3: content validity and reformulations

The Content Validity Index (CVI) was calculated for each item and for the overall scale. Most items showed an I-CVI of 1.00. One item containing a perspective verb, “The boy is winning the girl’s gift”, received an I-CVI of 0.57. All distractor images achieved I-CVI values above 0.83. For the assessment, the Scale-CVI (S-CVI) was 0.98 for correct answers and 0.99 for distractor distinctiveness. The test layout and final qualitative questionnaire were considered adequate (CVI = 0.86), whereas the test instructions required clarification (CVI = 0.71). Overall, considering instructions, layout, and the evaluator’s qualitative questionnaire, the scale achieved an S-CVI of 0.87.

In the qualitative analysis, judges’ comments and results were compiled and evaluated according to technical and theoretical criteria, with suggestions either accepted or discarded. Following an expert review, the image of the item with the lowest I-CVI was modified for clarity. The image was modified by adding a birthday hat to better identify the character receiving the gift. This revised image was approved by the test authors, resulting in a final TASComp version deemed satisfactory by both expert reviews and authors.

### 3.3 Steps 3 and 4: pilot study and final version

The pilot study indicated high acceptability and overall comprehension of the TASComp task. Participants demonstrated familiarity with the online platform and a clear understanding of instructions, regardless of age or prior technology experience. Acceptability was further supported by the absence of participant questions during the task and evaluators’ confirmation of the platform’s usability.

Performance data showed that most items were answered correctly, with a minimum accuracy rate of 83.33%. However, phonetic differentiation of responses, particularly for alternatives “B” and “D” in the online modality, occasionally compromised recording reliability, prompting procedural adjustments.

Based on these observations, the test instructions and administration manual were revised to improve procedural clarity and data accuracy. Specifically, to address phonetic ambiguities online, administrators were instructed to verbally confirm responses with participants when letters “B” or “D” were selected, ensuring correct recording and the validity of collected data.

### 3.4 Step 5: psychometrics properties of the online version of TASComp

In the last stage of the study, evidence of validity for the final computerized version of TASComp was investigated. The distribution of total correct answers for the entire test and in each block was nonparametric, with Shapiro–Wilk <0.001 for all blocks. The mean and standard deviation from each block (type of sentence) and for the total of the TASComp, was calculated in the two modalities of application, in-person and online, and for the total of participants (Table 3). Subsequently, a comparison of performance per type of sentence and the total score of the TASComp was conducted between in-person and online administration modalities, as demonstrated in Table 3.

**Table 3:** Comparison of performance by type of sentence across administration modalities (in-person and online) using independent samples *t*-test with BCa confidence interval (CI).

Type of sentence	Mean (*SD*)			Independent sample *t*-test			BCa 95% CI		
	Total (*n* = 102)	In person (*n* = 45)	Online (*N* = 57)	*t*	*p*	Cohens*’d*	*t*	Lower	Upper
Irreversible active	3.83 (0.38)	3.80 (0.41)	3.86 (0.35)	−0.80	0.43	−0.16	0.08	−0.21	0.10
Reversible active	3.83 (0.45)	3.80 (0.51)	3.86 (0.40)	−0.65	0.52	−1.33	0.09	−0.25	0.12
Perspective verb	3.85 (0.36)	3.84 (0.37)	3.86 (0.35)	−0.21	0.83	−0.04	0.07	−0.17	0.15
Irreversible passive	3.90 (0.330)	3.84 (0.42)	3.95 (0.23)	−0.78	0.15	−0.31	0.07	−0.25	0.03
Reversible passive	3.74 (0.47)	3.71 (0.51)	3.75 (0.43)	−0.46	0.65	−0.09	0.09	−0.23	0.13
Implausible passive	3.83 (0.42)	3.80 (0.55)	3.86 (0.35)	−0.67	0.50	−0.14	0.09	−0.25	0.11
Subject cleft	3.91 (0.29)	3.91 (0.29)	3.91 (0.29)	−0.02	0.98	−0.004	0.06	−0.11	0.10
Object cleft	3.52 (0.75)	3.67 (0.48)	3.40 (0.90)	1.77	0.06	−0.042	0.14	0.02	0.53
Right-branching subject relative	3.88 (0.32)	3.87 (0.34)	3.89 (0.31)	−0.43	0.67	−0.09	0.06	−0.17	0.10
Center- embedded subject relative	3.27 (0.88)	3.24 (0.93)	3.30 (0.84)	−0.30	0.76	−0.06	0.18	−0.49	0.33
Right-branching object relative	3.61 (0.66)	3.67 (0.52)	3.56 (0.76)	0.83	0.41	0.16	0.12	−0.14	0.35
Center- embedded object relative	3.25 (0.95)	3.29 (0.99)	3.21 (0.92)	0.41	0.68	0.08	0.19	−0.35	0.52
Total TASComp	44.45 (2.96)	44.44 (2.87)	44.46 (3.05)	−0.02	0.98	−0.004	0.59	−1.29	1.26

In general, all questions had a good proportion of correct answers, with a minimum of 3.25 points, which is the center embedded object relative block and maximum mean of 3.91 in the subject cleft sentences.

The analysis showed that most linguistic structures did not differ significantly between administration modalities (Table 3). The internal consistency analysis of the assessment, considering each question, was calculated using the Kuder–Richardson 20 coefficient (KR-20; Kuder and Richardson, 1937), obtaining a result of 0.66.

To evaluate validity evidence based on response processes, the impact of syntactic complexity on participants’ accuracy for each sentence type was assessed using paired-samples *t*-tests (Table 4). Regarding the distinction between object and subject extractions in relative clauses, a small-to-moderate effect was observed within right-branching sentences. In contrast, the comparison between subject and object clauses in the central position produced a negligible effect size that did not reach statistical significance. Also, a moderate effect size was observed in the comparison between subject and object cleft sentences. The comparison between right-branching and center embedded subject relatives yielded a moderate-to-large effect size, while the same positional comparison for object relative clauses resulted in a small-to-moderate effect. In passive structures, the results showed a significant difference between irreversible and reversible sentences, with a small-to-moderate effect size (Table 4).

**Table 4:** Paired samples *t*-test and Cohen’s d with BCa confidence intervals for syntactic complexity in TASComp by type of sentence.

Sentences	Paired samples *t*-test				BCa 95% CI			
	*M* difference (*SD*)	*t*	*p*	Cohen’s *d*	*t*	*Sig*. (2-tailed)	Lower	Upper
Irreversible active—reversible active	0 (0.55)	<0.001	1.00	0.00	0.06	1.00	−0.09	0.09
Irreversible passive—reversible passive	0.17 (0.55)	3.08	0.003	0.31	0.06	0.006*	0.06	0.28
Subject cleft—object cleft	0.39 (0.75)	5.30	<0.001	0.53	0.07	0.002*	0.26	0.53
Reversible passive—implausible passive	−0.10 (0.64)	−1.55	0.12	−0.15	−0.06	0.13	−0.21	0.02
Right-branching subject relative – Center-embedded subject relative	0.61 (0.90)	6.80	<0.001	0.67	0.09	<0.001**	0.46	0.77
Right-branching object relative – Center-embedded object relative	0.36 (1.04)	3.52	0.001	0.35	0.10	<0.001**	0.18	0.55
Right-branching subject relative – Right-branching object relative	0.28 (0.73)	3.78	<0.001	0.37	0.07	0.003*	0.15	0.41
Center-embedded subject relative – Center-embedded object relative	0.03 (1.12)	0.26	0.79	0.03	0.11	0.79	−0.18	0.24

*p < 0.05, **p < 0.001.

To examine evidence of validity based on relations to other variables, the TASComp total score was analyzed as the dependent variable in a beta regression model. Age and years of education were included as covariates, while the administration mode (in-person vs. online) was treated as a fixed factor. Model fit indices, parameter estimates, the Omnibus test, and marginal effects were subsequently evaluated. The suitability of the Generalized Linear Model was confirmed through an assessment of its goodness-of-fit indices. The model yielded favorable values (LogLikelihood = 172.31, AIC = −334.62, and BIC = −321.50), suggesting superior fit and parsimony compared to alternative models. Model deviance was 78.729 with 97 residual degrees of freedom. Additionally, the overdispersion indicator (χ^2^/df = 0.92) was near unity (1.00). This is a critical finding that validates the assumed variance structure and model specification, confirming the robustness of the fitted model for data analysis.

The Omnibus test revealed that the covariates years of study (χ^2^ (1) = 15.74, *p* < 0.001) and age (χ^2^ (1) = 7.98, *p* = 0.01) were statistically significant, demonstrating a substantial contribution to the model’s variance. In contrast, the main factor, Test Modality, did not reach statistical significance at this level of analysis (χ^2^ (1) = 0.27, *p* = 0.61; see Table 5).

**Table 5:** Parameter estimates of the model.

Variable	Effect	Estimate	SE	Exp(B)	Exp(B) 95% confidence intervals		*z*	*p*
					Lower	Upper		
(Intercept)	(Intercept)	2.53	0.08	12.53	11.02	14.98	30.92	<0.001
Age	Age	−0.21	0.07	0.81	0.70	0.94	−2.83	0.005
Test modality	Online—in person	−0.08	0.15	0.93	0.69	1.31	−0.52	0.606
Educational years	Educational years	0.06	0.02	1.06	1.02	1.10	3.97	<0.001

The marginal effect of age was −0.015, with a 95% confidence interval (CI) ranging from −0.03 to −0.004. For years of education, the effect was estimated at 0.004, with a 95% CI between 0.002 and 0.01. Finally, the effect of test modality (online vs. in-person) was −0.01, with a 95% CI ranging from −0.026 to 0.02.

**Figure 2:**
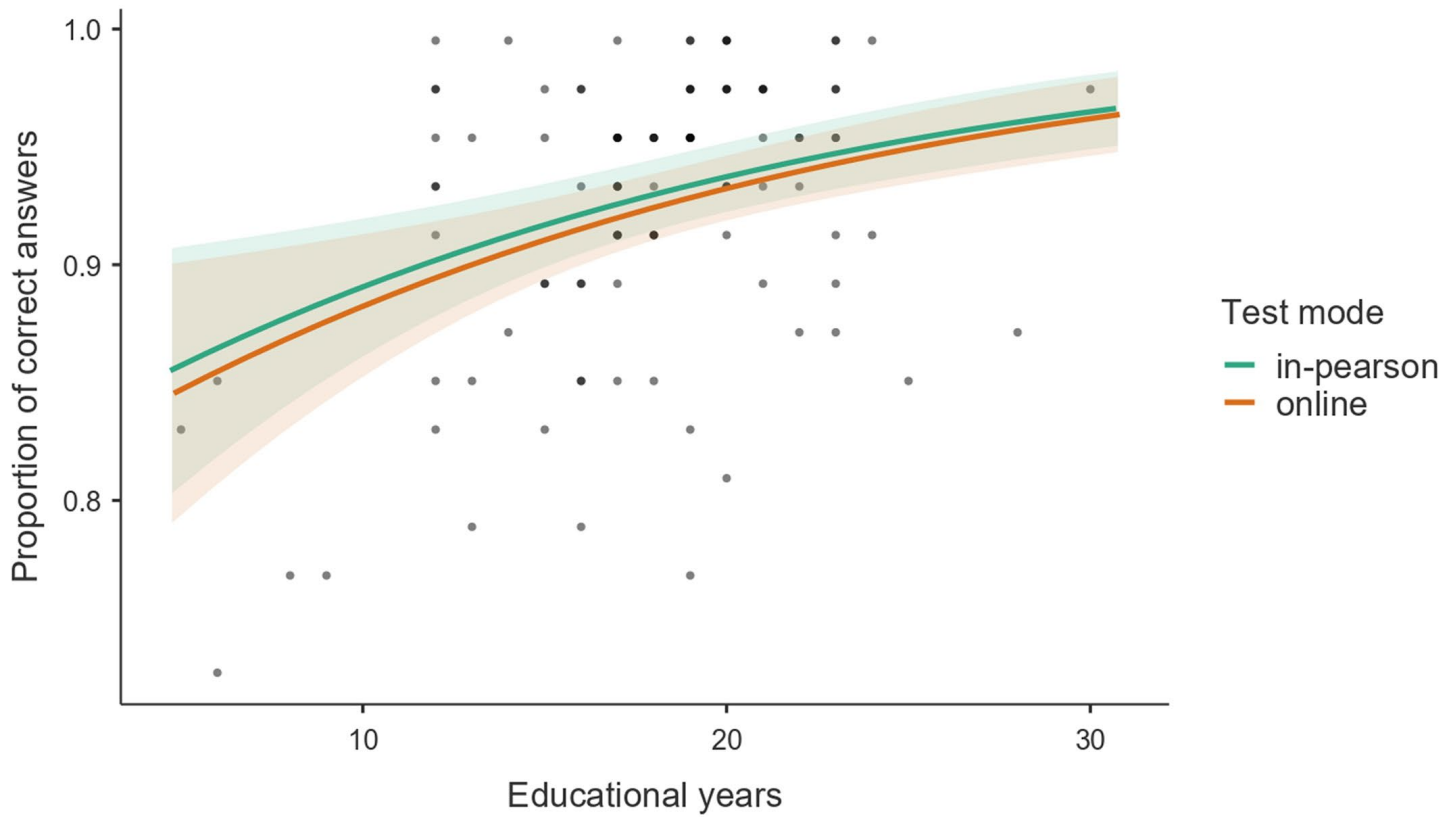
Proportion of correct answers in TASComp predicted by years of study, stratified by test modality.

Figure 2 presents a scatter plot of the observed proportion of correct answers in the TASComp as a function of years of study, overlaid with predicted values from the beta regression model. The individual data points illustrate the distribution of participant scores across the educational continuum. The fitted regression lines for both the in-person and online modalities indicate a consistent upward trend, showing that as years of study increase, the proportion of correct answers also rises. The substantial overlap of the 95% confidence intervals and the close proximity of the regression lines across the observed data points further demonstrate that the administration modality did not significantly alter the relationship between education and test performance.

**Figure 3:**
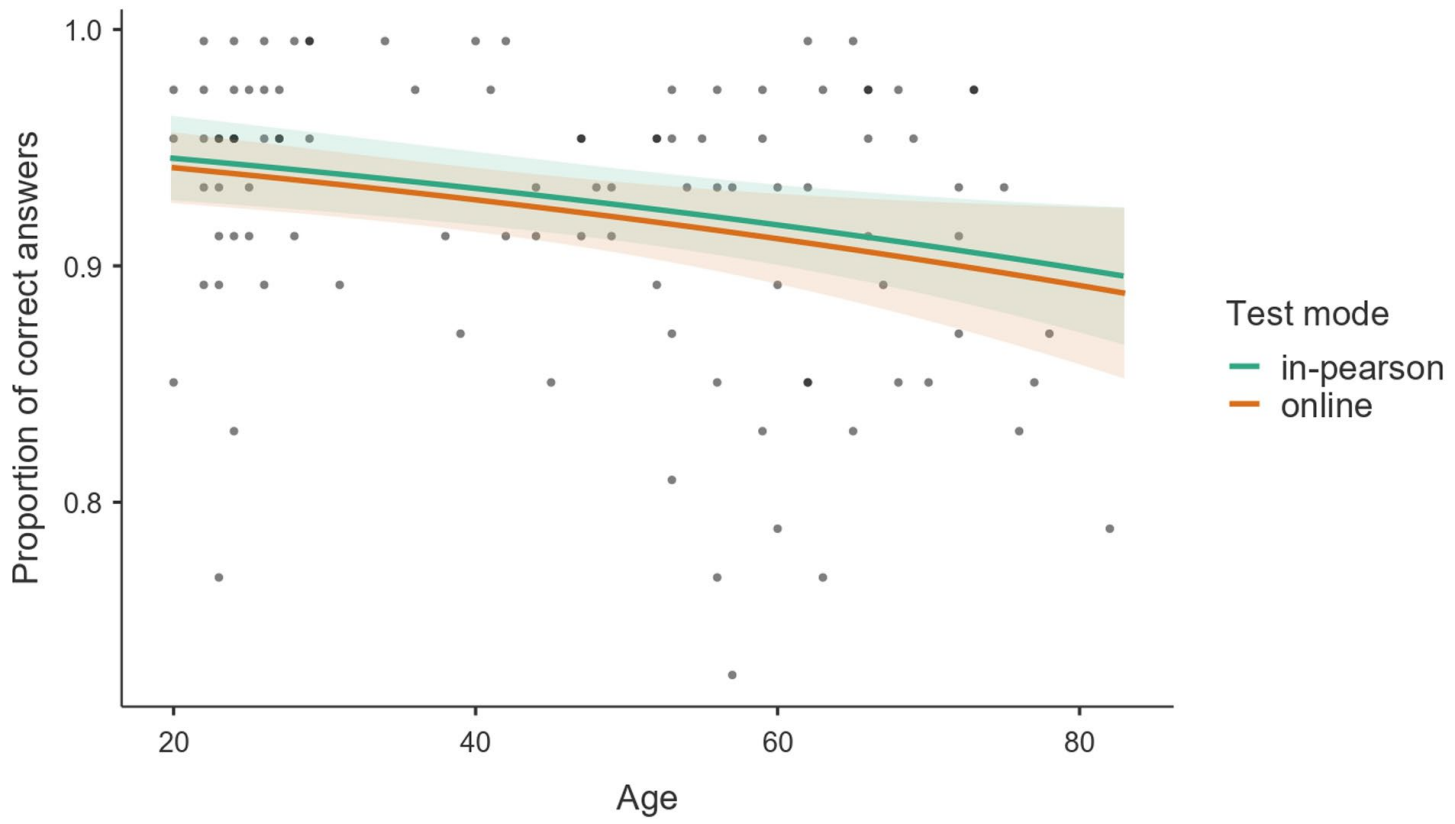
Proportion of correct answers in TASComp predicted by age, stratified by test modality.

In addition, Figure 3 presents a scatter plot of the observed scores alongside fitted regression lines illustrating the relationship between age and TASComp performance. The data points reveal a negative correlation, where advancing age predicts a decrease in total test accuracy. Notably, the regression lines for both the in-person and online modalities follow nearly identical downward trajectories, consistent with the stability observed in the analysis of years of study. This demonstrates that the administration format does not significantly modulate the impact of age on test performance, as evidenced by the substantial overlap of confidence intervals.

Consistent with these findings, post-hoc pairwise comparisons (using Bonferroni and Tukey adjustments) revealed that the difference between the in-person and online modalities was not statistically significant, with an estimated mean difference of 0.01 (SE = 0.01, *z* = 0.52, *p* = 0.60).

## 4 Discussion

The results of the computerized TASComp adaptation demonstrated satisfactory usability and robust psychometric properties, supporting its suitability for use with both young and older adults, with potential applicability in clinical populations. The TASComp demonstrated strong content validity, with most items and distractors exceeding the 0.83 threshold (Lynn, 1986). These results confirm that the images provided unambiguous visual information of the target sentences. Notably, the high validity of the distractors ensures the task minimizes false positives caused by visual confusion. By adhering to strict construction protocols to avoid visual errors (Ferrari, 2022), the TASComp ensures that participant performance reflects linguistic competence, lexical and syntactic, rather than visual decoding skills, an important requirement for the adequacy of a sentence comprehension language assessment. Thus, the high CVI scores attest to the test’s adequacy, establishing it as a reliable tool for assessing language processing without the interference of construct-irrelevant information. Conversely, one item involving a perspective-taking verb showed comparatively lower agreement among experts, failed to meet the adequacy criteria. The original sentence in Brazilian Portuguese was “O rapaz está ganhando o presente da moça.” (The boy is receiving a gift from the girl), that was subsequently altered by the authors for improved clarity. This isolated deficit highlights the limitation of the image in capturing semantical relational concepts, suggesting that perspective-dependent verbs require more explicit visual cues to ensure validity. Therefore, the results demonstrating the evidence of content validity of TASComp (American Educational Research Association et al., 2014).

The adaptation to a computerized platform (Stoet, 2016) enabled effective participant interaction with instructions and stimulus presentation, while also supporting examiner usability by facilitating response recording and integrating stimuli without an auxiliary protocol. Response selection by the administrator remains challenging due to the need for standardized administration and accurate recording; however, no issues were observed after revising the instructions, confirming the test’s applicability for teleneuropsychological use. This finding represents a notable advance, addressing a critical gap in Brazil due to the limited availability of validated instruments for language assessment, particularly computerized tools for evaluating sentence processing.

The prior conduct of the pilot study was decisive in ensuring this applicability in the final stage, allowing for the precise adjustment of instructions and enabling the standardization of procedures. This methodological approach confirms the crucial importance of adapting psychological instruments following rigorous and systematic steps to ensure the technical quality and validity of the measure, as recommended by international standards (American Educational Research Association et al., 2014).

Descriptive analyses revealed high accuracy rates, reflecting the test’s accessibility for a non-clinical sample of healthy adults. This performance demonstrates the overall adequacy of the assessment; given the expectation of preserved language comprehension in this population, observed errors are likely attributable to executive processing limitations rather than primary linguistic deficits (Caplan et al., 2016; Poulisse et al., 2019; Sung et al., 2017). With regards to internal consistency, the Kuder–Richardson 20 (KR-20; Kuder and Richardson, 1937) index indicated a marginal result. However, this measure should be interpreted in light of the nature of the data. The psychometric literature (Streiner, 2003) warns that reliability coefficients are directly dependent on the variance of the scores. Given the homogeneity of the sample, consisting in healthy adults and with high educational level, and the dichotomous nature of the items (correct or incorrect answer), the observed variance restriction (ceiling effect) tends to underestimate internal consistency, not necessarily indicating flaws in the reliability of the instrument. However, internal consistency should be verified in a heterogeneous sample, which also interferes with the heterogeneity of the scores.

Validity based on response processes for the TASComp was supported by comparisons of mean accuracy between online and in-person groups, which showed no significant differences across nearly all linguistic conditions. These findings are consistent with recent teleneuropsychology literature, indicating that properly adapted computerized language assessments can yield results comparable to traditional paper-and-pencil administration (Brown et al., 2024; Chapman et al., 2020; Krynicki et al., 2023). Therefore, computerized tests can be considered psychometrically valid, offering a reliable and valid mechanism for assessing linguistic and cognitive functions, with results consistent with the established normative data (American Educational Research Association et al., 2014; Brown et al., 2024). These results support the suitability of TASComp for online administration.

Regarding the final version, evidence of validity based on response processes was examined by comparing the mean accuracy obtained in each syntactic structure. These results aligned with psycholinguistics literature, confirming the TASComp’s sensitivity to the cognitive demands imposed by varying levels of sentence complexity. The results showed that participants performed significantly better on irreversible passive sentences than on reversible passive sentences. This result can be interpreted considering that the passive voice represents a deviation from the canonical Subject-Verb-Object order (Corrêa et al., 2017; Townsend and Bever, 2001). This reconfiguration imposes a high cognitive demand, as it forces the processor to inhibit the standard heuristic expectation that the syntactic subject, especially when animate, is the performer of the action (Lima Júnior and Corrêa, 2015). The advantage of irreversible passives suggests that, given this structural complexity, the cognitive system relies on lexical-semantic clues (the meaning of words restricting the action) to facilitate understanding. In contrast, in reversible passive sentences, where both nouns are animate and plausible as agents, this semantic route is not available, making comprehension dependent exclusively on algorithmic syntactic processing, which increases the error rate (Ferreira, 2003; Ferreira et al., 2001). The absence of a significant difference between irreversible and reversible active sentences corroborates this hypothesis, indicating that, in the active voice (frequent canonical form), the efficiency of syntactic processing in healthy adults is robust enough to render semantic cues redundant.

In cleft sentences, subject structures perform significantly better than object structures. This result is in line with theoretical processing models, considering that cleft sentences impose specific cognitive demands due to the syntactic movement used to focus on an element of the sentence (Alves et al., 2015; Lambrecht, 2001). While subject cleft sentences preserve a shorter distance between the focused element and its original trace, facilitating integration, object cleft sentences require the processing of long-distance dependencies with the presence of an intervening element (the subject of the relative clause). This structural configuration of object clefts substantially increases the load on working memory and requires greater recruitment of executive functions, specifically inhibitory processes, necessary to select the correct focus and inhibit alternative characters in the action (Rodrigues et al., 2024). In this case, processing difficulties can be identified in healthy adults, given the greater demand on cognitive resources.

Analyses showed that right-branching subject relative clauses were processed with significantly greater accuracy than center-embedded subject relative clauses. The same pattern was observed in object structures, where right-branching Object Relatives performed better than Center-embedded Object Relatives. Theoretically, relative clauses demand high cognitive cost because they involve the movement of a constituent, creating a syntactic dependency between the antecedent and the gap left in the original position (Forster and Corrêa, 2016). The systematic advantage of right-branching structures, regardless of whether they are subject or object, is explained by the maintenance of syntactic linearity, which avoids interruption of the main clause and reduces the load on working memory (Gibson et al., 2005). In contrast, the center embedding imposes a critical overload by interrupting the processing of the main clause, forcing the cognitive processing system to keep the subject of the main clause active in memory while processing the entire subordinate structure. This structural “interruption” cost proved to be so significant that a direct comparison between the two most complex forms (Center embedded Subject Relative vs. Center embedded Object Relative) did not result in a significant difference, suggesting that when the embedding is central, the memory overload reaches a threshold that equates the difficulty (Friedmann et al., 2009; Gibson, 1998; Rodrigues et al., 2024).

However, manipulation of semantic plausibility in complex structures did not generate performance differences between semantically plausible and implausible passive reversible sentences. This finding can be interpreted considering the demographic characteristics of the sample, composed mainly of young adults with high educational attainment and no linguistic deficits, unlike what might occur in clinical populations, where semantic violation tends to compete with syntactic analysis and generate errors (Christianson et al., 2006; Ferreira et al., 2002), the healthy adults in this sample demonstrated sufficient cognitive resources to inhibit semantic strangeness and sustain correct structural analysis.

Evaluation of evidence of validity based on relations to other variables indicated that years of education and age are significant predictors of TASComp performance. This aligns with prior research highlighting formal education as a key determinant of performance in linguistic tasks (Akashi and Ortiz, 2018; Pereira and Ortiz, 2022). Reading and writing habits may contribute to cognitive reserve, supporting comprehension of complex sentences even in older adults (Malcorra et al., 2022; Montemurro et al., 2021; Riffo et al., 2025). Education thus serves as a protective factor, with higher educational attainment associated with better working memory, executive function, and attentional resources, cognitive abilities essential for processing complex syntactic structures (Malcorra et al., 2022).

Also, age proved to be a significant negative predictor, showing a marginal decline in the proportion of correct answers for each year of the participant’s age. This result is consistent with theory models that link aging to a reduction in working memory capacity that affects the processing of complex sentences (Poulisse et al., 2019). This corroborates that syntactic deficits are sensitive markers not only in aphasia but also in cognitive decline associated with aging and in early stages of neurodegenerative disorders (Caplan et al., 2016; Gilardone et al., 2024; Ivanova and Dronkers, 2022; Kaltsa et al., 2024; Murray, 2018; Sung et al., 2017, 2020).

Finally, the modality of the test application (in-person or online) did not yield a significant effect, meaning it was not a predictor of the total TASComp score. This finding aligns with the previous comparison of mean results between the groups for each sentence type. The *post hoc* analysis corroborated this equivalence, revealing a negligible and insignificant mean difference between the groups. These findings further ensure that the test maintains its psychometric accuracy regardless of the means of administration, reinforcing what is expected by the literature, namely that the administration of standardized tests in teleneuropsychology does not interfere with patient results compared to in-person administration (Brown et al., 2024; Chapman et al., 2020; Krynicki et al., 2023). Consequently, these results corroborate the feasibility of TASComp for use in teleneuropsychology, a field that has become essential for expanding access to cognitive assessment (Alva et al., 2025; Serrano-Juárez et al., 2023).

## 5 Conclusion

The present study confirms that the TASComp is a robust instrument for assessing sentence processing, with a design that effectively captures the nuances of linguistic comprehension with different types of sentences. Although the internal consistency observed was moderate, this result must be interpreted considering the sample’s homogeneity and the ceiling effects typical of healthy adults; it does not diminish the task’s value as a valid measure of language competence. Importantly, the instrument distinguished varying levels of processing difficulty, consistent with psycholinguistic models that integrate syntactic complexity and working memory demands.

Furthermore, the analysis established the significant impact of age and education on syntactic processing, demonstrating that even healthy individuals without language disorders exhibit distinct performance patterns based on these demographic variables. This finding highlights the critical need to develop stratified norms to prevent diagnostic errors and ensure accurate interpretation across different life stages and socioeconomic backgrounds. Consequently, these results also reinforce that language assessment must use different types of sentences, moving towards a deep investigation of complex grammatical and semantic structures. Such an approach is essential for detecting subtle deficits across diverse populations (Kaltsa et al., 2024; Thothathiri et al., 2023; Wallace et al., 2023).

Finally, regarding the mode of administration, the findings underscore the viability of standardizing tests for remote use. The TASComp demonstrated favorable usability in the online environment without compromising the instrument’s robustness, as evidenced by the stability of scores across modalities. This confirms its suitability for teleneuropsychology, expanding access to assessment in diverse settings. Ultimately, the TASComp could provide important information in language processing across different populations, by such in-depth assessment is indispensable for designing rehabilitation programs, as the precise identification of syntactic deficits provides the necessary data to develop personalized, evidence-based therapeutic strategies.

### 5.1 Limitations and future studies

This study has some limitations. The sample included only healthy adults, with relatively few older participants compared to younger adults, and was highly educated, limiting the analysis of variability in items and blocking scores by syntactic complexity. Future research should include larger and more diverse samples to allow for a more thorough examination of item difficulty and discrimination, as well as comparisons between healthy individuals and populations with language comprehension deficits, such as individuals with aphasia, neurological impairments, older adults, or patients with neurodegenerative conditions.

Regarding future studies, the development of a reduced version of the test is being considered. The abbreviated version aims to facilitate screening for syntactic difficulties by focusing on the most discriminative items. Additionally, future research will compare performance between clinical and control groups and conduct a qualitative analysis of errors to distinguish random mistakes from systematic syntactics or other deficits. These investigations will also allow examination of relationships with cognitive functions, such as the influence of working memory and inhibitory control on performance across different sentence types.

## Data Availability

The raw data supporting the conclusions of this article will be made available by the authors, without undue reservation.
